# Ascertaining gene flow patterns in livestock populations of developing countries: a case study in Burkina Faso goat

**DOI:** 10.1186/1471-2156-13-35

**Published:** 2012-05-07

**Authors:** Amadou Traoré, Isabel Álvarez, Iván Fernández, Lucía Pérez-Pardal, Adama Kaboré, Gisèlle MS Ouédraogo-Sanou, Yacouba Zaré, Hamidou H Tambourá, Félix Goyache

**Affiliations:** 1 INERA, 04 BP 8645, Ouagadougou 04, Burkina Faso; 2 SERIDA-Deva, C/Camino de Rioseco 1225, E-33394, Gijón (Asturias), Spain; 3 Laboratoire National d’Elevage, 01 BP 7021, Ouagadougou 01, Burkina Faso

## Abstract

**Background:**

Introgression of Sahel livestock genes southwards in West Africa may be favoured by human activity and the increase of the duration of the dry seasons since the 1970’s. The aim of this study is to assess the gene flow patterns in Burkina Faso goat and to ascertain the most likely factors influencing geographic patterns of genetic variation in the Burkina Faso goat population.

**Results:**

A total of 520 goat were sampled in 23 different locations of Burkina Faso and genotyped for a set of 19 microsatellites. Data deposited in the Dryad repository: http://dx.doi.org/10.5061/dryad.41h46j37. Although overall differentiation is poor (F_ST_ = 0.067 ± 0.003), the goat population of Burkina Faso is far from being homogeneous. Barrier analysis pointed out the existence of: a) genetic discontinuities in the Central and Southeast Burkina Faso; and b) genetic differences within the goat sampled in the Sahel or the Sudan areas of Burkina Faso. Principal component analysis and admixture proportion scores were computed for each population sampled and used to construct interpolation maps. Furthermore, Population Graph analysis revealed that the Sahel and the Sudan environmental areas of Burkina Faso were connected through a significant number of extended edges, which would be consistent with the hypothesis of long-distance dispersal. Genetic variation of Burkina Faso goat followed a geographic-related pattern. This pattern of variation is likely to be related to the presence of vectors of African animal trypanosomosis. Partial Mantel test identified the present Northern limit of trypanosome vectors as the most significant landscape boundary influencing the genetic variability of Burkina Faso goat (*p* = 0.008). The contribution of Sahel goat genes to the goat populations in the Northern and Eastern parts of the Sudan-Sahel area of Burkina Faso was substantial. The presence of perennial streams explains the existence of trypanosome vectors. The South half of the Nakambé river (Southern Ouagadougou) and the Mouhoun river loop determined, respectively, the Eastern and Northern limits for the expansion of Sahelian goat genes. Furthermore, results from partial Mantel test suggest that the introgression of Sahelian goat genes into Djallonké goat using human-influenced genetic corridors has a limited influence when compared to the biological boundary defined by the northern limits for the distribution of the tsetse fly. However, the genetic differences found between the goat sampled in Bobo Dioulasso and the other populations located in the Sudan area of Burkina Faso may be explained by the broad goat trade favoured by the main road of the country.

**Conclusions:**

The current analysis clearly suggests that genetic variation in Burkina Faso goat: a) follows a North to South clinal; and b) is affected by the distribution of the tsetse fly that imposes a limit to the Sahelian goat expansion due to their trypanosusceptibility. Here we show how extensive surveys on livestock populations can be useful to indirectly assess the consequences of climate change and human action in developing countries.

## Background

Desertification limits the presence of vectors of trypanosomosis and, therefore, favours the introgression of Sahelian genes into southern trypanotolerant livestock populations in West Africa [1-3]. This process is diluting the genetic background of trypanotolerant African livestock, which result from a unique process of natural adaptation [4,5]. Burkina Faso is a landlocked country located in the limit between the Sahel and the southern-humid zones of West Africa. Due to its geographic location, it has been considered as a country of choice to study the effects of desertification on livestock populations [1].

A detailed description of the geography, environmental areas and goat populations of Burkina Faso is given as Supplementary Background. Burkina Faso is a flat country with no clear geographic barriers limiting the spreading of animal populations. Only the Mouhoun river (formerly known as Black Volta) has perennial streams (see Additional file 1).

Three main environmental areas can be defined in Burkina Faso according to climate conditions and types of vegetation [6-8] (see Additional file 1): a) the arid Sahel area, covering the Northern part of Burkina Faso; b) the Sudan area, covering the Southern part of Burkina Faso with annual rainfall higher than 900 mm; and c) the Sudan-Sahel area, covering the central part of the country and with very variable rainfall. The droughts of the 1970s and 1980s led to a shift southwards of the limits of these environmental areas.

Each of the environmental areas of Burkina Faso is assumed to be the habitat of a different goat population [9,10]: the Sahelian goat population is the Burkina Faso representative of the African long-legged goat group, spread throughout the Sahel region of West Africa; and the Djallonké population, located in the Sudan area of Burkina Faso, is a short-eared and small-horned goat also known as West African Dwarf goat. The Djallonké goat are usually considered trypanotolerant while Sahelian goat is trypanosusceptible [5,11]. A transition population between these two major breeds (the Mossi goat) is kept in the Central Sudan-Sahel area. The Mossi goat is considered the northernmost representative of the trypanotolerant West African Dwarf goat population in Burkina Faso [9,10]. However, Mossi goat are considered to be less trypanotolerant and have differential characteristics with Djallonké goat due to the particularly arid ecosystem in which it is spread and to a sustained introgression of Sahelian goat genes [9].

The Djallonké goat, living in tsetse endemic areas, are known to be more resistant to trypanosomosis than breeds living in tsetse free areas [11-13]. Although Djallonké goat should be considered as resilient rather than resistant to trypanosomosis [11], it has been suggested that Djallonké goat possess an innate ability to acquire immunity in scenarios of repetitive trypanosome infections [11,13]. Therefore, introgression of the Sahelian livestock genes into South may be limited by the presence of vectors of trypanosomosis (tsetse flies; *Glossina spp*.) [5,11,14]. The northwestern tsetse distribution limits in 2009 [14] started above the Mouhoun river loop to shift southwards following the course of the river Mouhoun ( Additional file 1). Presence of tsetse flies in the South-East Burkina Faso is limited to the southern part of the Nakambé river and protected environmental areas near the Togo border ( Additional file 1). Trypanosusceptibility could restrict the possibilities of gene flow from Sahelian goat into the southernmost Burkina Faso herds kept in favourable environments for the trypanosome vectors [1]. The assessment of such scenarios is important since African animal trypanosomosis is a major obstacle to the development of more efficient and sustainable livestock production systems [14].

A previous study using a limited number of samples and focusing on the assessment of the between-breeds genetic relationships in Burkina Faso goat [10], suggested that Burkina Faso goat is a poorly differentiated animal population with significant gene flow between environmental areas. This lack of differentiation was probably due to the fact that African livestock breeds are mainly defined from the farmholding ethnic groups or geographic areas into which the individuals are found [10,15-17]. Consistency between neutral molecular information and those criteria used for definition of livestock breeds could be low when no selection programmes exist and long-distance livestock trading is intense.

Studies on how landscape features influence genetic structure and gene flow patterns are frequent in natural populations [18,19]. However, in livestock populations the analyses of geographic patterns of genetic variation is mainly focused on the ascertainment of historical genetic events related to domestication [17,20]. The current research starts from our previous study [10] and the current knowledge on the relationships between the Sahelian and the Djallonké goat at the Sahel region level [11]. We considered the Burkina Faso goat population as a whole and assumed the possible existence of: a) two original goat populations, Djallonké and Sahelian, differing from trypanosome tolerance; b) a gradient of introgression of Sahel genes into Djallonké goat favoured by livestock trading [9]. The main aim of this study is to ascertain if there exists differences in the genetic background of the Burkina Faso goat and if these differences are consistent with geographic location, tsetse distribution or human action. An extensive survey of the genetic variability of the Burkina Faso goat was carried out. A total of 520 goat, sampled in 23 different locations, were genotyped for 19 microsatellite markers. Molecular information will be compared to published information on distribution of trypanosome vectors in the Burkina Faso territory.

## Results

The parameters F_ST_, F_IS_ and F_IT_ estimated for the whole dataset were, respectively, 0.067 ± 0.003, 0.035 ± 0.007 and 0.100 ± 0.007 illustrating a scenario with moderate differentiation and heterozygote deficiency. Overall expected heterozygosity was moderate (0.575 ± 0.003). This tended to be lower in the populations sampled in the Sudan area (Table 1). Also, the Sudan populations had the lowest ‘rarefacted’ number of alleles per locus.

**Table 1:** Description of sampling

**Environmental**	**Population**			**Coordinates**		**Genetic parameters**				**PCA**	**Admixture coefficients**	
area	Number^a^	name	N	latitude	longitude	H_e_	F_IS_	k	k_(6)_	Factor1	LEADMIX	LEA
Sahel	1	Fadar-Fadar	24 (7)	15°01'30.81"N	0°13'60.00"W	0.475 (0.017)	0.044 (0.035)	4.8	2.6	0.347	0.997^a^	0.979
	2	Gorom-Gorom	25 (11)	14°26'60.00"N	0°13'60.00"W	0.565 (0.013)	0.005 (0.031)	5.5	2.9	0.394	0.999^a^	0.843
	3	Yakouta	22 (18)	14°04'60.00"N	0°07'60.00"W	0.587 (0.010)	0.044 (0.026)	6.2	3.0	0.271	0.996^a^	0.941
	4	Dori	11 (5)	14°01'59.88"N	0°40'58.84"W	0.584 (0.012)	−0.054 (0.027)	4.9	2.9	0.598	0.992^a^	0.904
	5	Katchari	14 (2)	13°55'05.49"N	0°17'05.97"E	0.531 (0.011)	−0.067 (0.032)	4.5	2.8	0.568	1.000^a^	0.933
	6	Tougouri	27 (14)	13°18'54.76"N	0°31'05.07"W	0.551 (0.016)	0.100 (0.036)	5.6	2.8	0.322	0.877^a^	0.802
	7	Yalgo	26 (13)	13°28'00.00"N	1°33'00.00"W	0.572 (0.015)	0.047 (0.031)	5.8	2.9	0.331	0.999^a^	0.766
	8	Kaya	23 (12)	13°04'60.00"N	1°04'60.00"W	0.553 (0.017)	0.037 (0.035)	6.1	2.9	0.183	0.797	0.594
Sudan-Sahel	9	Ziniaré	27 (13)	12°34'60.00"N	1°18'00.00"W	0.516 (0.014)	0.018 (0.035)	5.6	2.7	−0.057	0.699	0.589
	10	Ouagadougou	23 (11)	12°21'52.69"N	1°32'01.91"W	0.527 (0.012)	0.054 (0.036)	5.1	2.7	−0.045	0.495	0.410
	11	Solenzo	28 (13)	12°10'60.00"N	4°04'60.00"W	0.558 (0.014)	0.054 (0.030)	5.7	2.8	−0.234	0.465	0.338
	12	Fada N’Gourma	28 (13)	12°04'00.00"N	0°21'00.00"E	0.537 (0.013)	0.005 (0.030)	5.8	2.8	0.066	0.631	0.458
	13	Sabou	29 (9)	11°45'00.00"N	3°30'60.00"W	0.525 (0.013)	0.024 (0.040)	5.3	2.7	−0.101	0.496	0.210
	14	Pabré	10 (1)	12°30'00.00"N	1°34'00.00"W	0.555 (0.012)	0.025 (0.024)	4.8	2.9	−0.120	0.488	0.404
	15	Saponé	15 (12)	12°03'10.00"N	1°36'13.00"W	0.509 (0.015)	0.124 (0.047)	4.7	2.6	−0.209	0.250	0.482
	16	Kamboinsé	9 (3)	12°03'00.00"N	1°31'00.00"W	0.581 (0.013)	0.002 (0.032)	4.8	3.1	−0.445	0.482	0.592
	17	Boromo	23 (7)	11°45'00.00"N	2°55'60.00"W	0.525 (0.016)	0.053 (0.032)	5.3	2.8	−0.123	0.422	0.500
	20	Bittou	29 (14)	11°15'00.00"N	0°17'60.00"W	0.528 (0.014)	0.029 (0.028)	5.2	2.8	−0.078	0.361	0.456
Sudan	18	Bobo Dioulasso	33 (9)	11°29'17.77"N	3°31'04.14"W	0.544 (0.015)	0.038 (0.035)	5.8	2.8	−0.110	0.542	0.631
	19	Houndé	22 (0)	11°27'03.84"N	4°27'09.35"W	0.526 (0.011)	−0.022 (0.042)	4.5	2.6	−0.182	0.062^b^	0.267
	21	Orodara	28 (15)	10°58'25.49"N	4°54'29.06"W	0.502 (0.016)	0.104 (0.039)	5.5	2.6	−0.290	0.004^b^	0.059
	22	Gaoua	21 (10)	10°19'29.93"N	3°10'25.35"W	0.477 (0.020)	0.054 (0.046)	4.7	2.6	−0.188	0.000^b^	0.026
	23	Kampti	23 (8)	10°07'60.00"N	3°27'00.00"W	0.497 (0.023)	0.120 (0.040)	5.3	2.6	−0.240	0.000^b^	0.009
TOTALS			520 (220)			0.575 (0.003)	0.035 (0.007)	11.9	3.0			

Sample size (N; number of males in brackets), geographic coordinates, expected heterozygosity (H_e_; s.d. in brackets), heterozygote deficiency within subpopulation (F_IS_; s.d. in brackets), raw (k) and ‘rarefacted’ (k_(6)_) average number of alleles per locus per population are given. The component scores corresponding to the Factor 1 identified via Principal Component Analysis (PCA) and the admixture coefficients computed using the programs LEADMIX and LEA (see text) are also given.

^a^numbers attached to each population are consistent with those shown in the Figures and roughly inform on the between-populations differences in latitude (the lower the number the northerly the location).

^b^upper 95% Confidence Interval bound higher than 1.0.

^c^lower 95% Confidence Interval bound lower than 0.0001.

**Figure 1:**
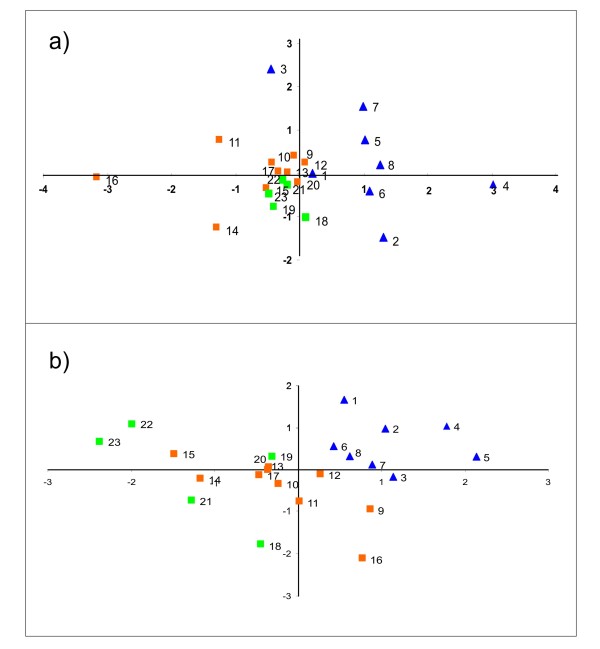
** Bidimensional scaling plots constructed using the complementary of the between-populations molecular coancestry matrix (1 -*f***_***ij***_**; Plot a) and the between-populations Reynolds’ distance matrix (*D***_***R***_**; Plot b).** Populations sampled in the Sudan, Sudan-Sahel and Sahel areas are, respectively, in green squares, orange squares and blue triangles. Numbers are consistent with those listed in Table 1 for the sampled populations.

Figure 1 summarises the between-populations genetic relationships (see also Additional file 2: Table S1). Bidimensional scaling plots constructed using the complementary of the between-populations molecular coancestry matrix (1 - *f*_*ij*_) and the between-populations *D*_*R*_ matrix gave complimentary information. On Dimension 1 (X-axis) of both distances, the populations sampled in the Sahel area are differentiated from the others. Plot 2a does not provide a clear differentiation between the Sudan and the Sudan-Sahel populations. Molecular coancestry partially reflects the between-populations genetic identity [21]. The *D*_*R*_ plot (Figure 1b) tends to reflect a Northeast-Southwest gradient of variation with no clear differentiation between the most northerly and most easterly Sudan-Sahel populations (9, 11, 12) and the Sahel goat populations. Note that, in this plot, the Sudan population 18 (Bobo Dioulasso) is a clear exception of this pattern.

**Figure 2:**
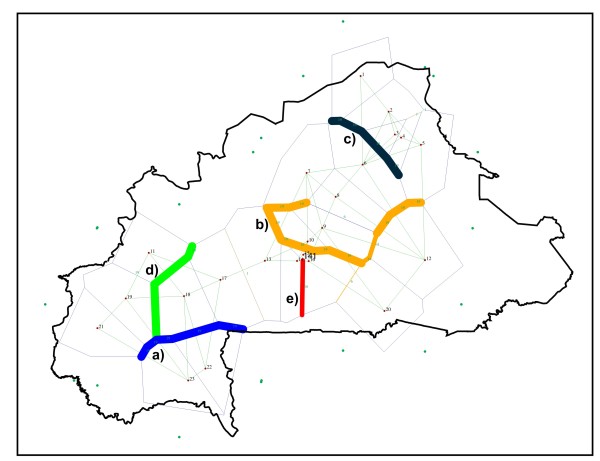
** Most likely genetic discontinuities identified in Burkina Faso goat using the program Barrier.** Barrier a) (in blue) separates population 18 (Bobo Dioulasso) from populations 22 and 23; barrier b) (in orange) separates populations southern Ouagadougou (population 10) from the others; barrier c) (in black) separates most populations sampled in the Sahel area from the others; barrier d) (in green) separates population 18 from populations sampled in eastern Sudan area (19 and 21) and population 11; barrier e) (in red) separates population 20 from the other populations sampled in southern latitudes in Burkina Faso. Numbers are consistent with those listed in Table 1 for the sampled populations.

In a landscape with no clear geographic barriers for livestock movement, we applied the Monmonier’s Maximum-difference algorithm [22], as implemented in the program Barrier version 2.2 [23], to identify possible genetic boundaries. Five likely genetic boundaries, zones where genetic differences between pairs of populations are the highest, were identified (Figure 2). Boundaries a) and d) separated population 18 (Bobo Dioulasso) from the other populations in the Sudan area. Boundaries b) and e) showed the existence of genetic differences in the populations sampled in the central (Southern Ouagadougou; population 10) and south east Burkina Faso (separating population 20 from the populations sampled in the Sudan area). Boundary c) separated the “pure” Sahelian goat from the rest of the sampled populations.

PCA allowed the identification of 23 different factors explaining 100% of the variability in the dataset. One factor (eigenvalue = 21.03) explained most genetic variability (91.44%) in Burkina Faso goat. The other factors had eigenvalues lower than 1 and, therefore, were not used for subsequent analyses. Factor 1 scores computed for each population are given in Table 1. Component scores computed for the Sahel populations are positive while those for the Sudan-Sahel and Sudan populations were negative except for that of the eastern population 12 (Fada N’Gourma).

Table 1 also gives the relative contributions of the Sahel goat to each population computed using the programs LEADMIX [24] and LEA [25]. Overall, both methods gave consistent results. Note that, using LEADMIX, 95% confidence interval of the estimates computed for the populations pooled to construct the Sahel and the Djallonké populations are out of the parametric space. This also happened with the estimates of populations 6 and 7 showing that they would have similar genetic background to “pure” Sahel populations (from 1 to 5). Both the LEADMIX and the LEA scores computed for population 18 (Bobo Dioulasso) were higher than expected, suggesting a significant introgression of Sahelian goat genes into this Southern location.

**Figure 3:**
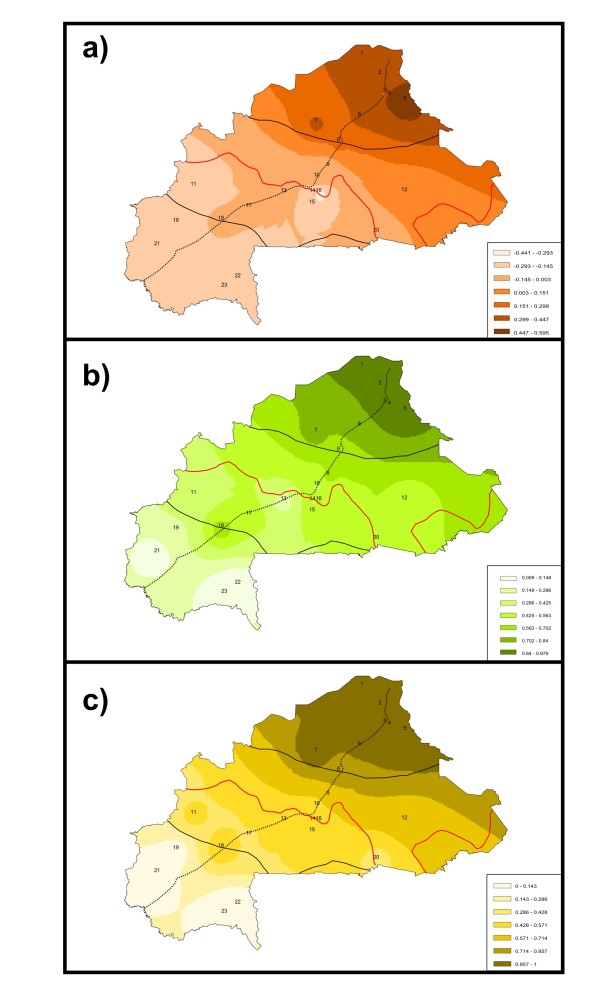
** Maps illustrating patterns of genetic variation in Burkina Faso goat.** Maps **a**), **b**) and **c**) are synthetic maps illustrating, respectively, geographic variation of the first factor identified using principal component analyses (PCA), and the relative parental contributions from Sahelian goat for each of the 23 sampled populations as determined using the programs LEADMIX (Map **b**) and LEA (Map **c**). To make the interpretation of the maps easier, the present limits of the three environmental areas (Sahel, in the North, Sudan, in the South, and central Sudan-Sahel area; solid black lines), the Northern tsetse limit in Burkina Faso reported in 2009 [14] (solid orange lines) and the main road of Burkina Faso (Dori-Ouagadougou-Bobo Dioulasso; populations 4, 10 and 18; dotted line) are also illustrated.

Population scores computed using PCA, LEADMIX and LEA were used to construct synthetic maps illustrating geographic genetic variation in Burkina Faso goat (Figure 3). The three Maps consistently allowed to assess that: i) the contribution of Sahel goat genes to the goat populations in the North (6, 7, 8) and the East (population 12) of the Sudan-Sahel area is substantial; ii) the perennial streams of the South half of the Nakambé river (Southern Ouagadougou) and the Mouhoun river loop explain, respectively, the Eastern and Northern limits of the expansion of tsetse flies and, therefore, of the Sahelian goat genes (see also Figure 2 and Additional file 1); and iii) a secondary introgression of the Sahelian goat genes southwards into the Sudan-Sahel and Sudan areas may follow the main road from Ouagadougou (population 10) to Bobo Dioulasso (population 18) leading to a genetic differentiation within the populations sampled in the Sudan area (see also Figure 2).

**Figure 4:**
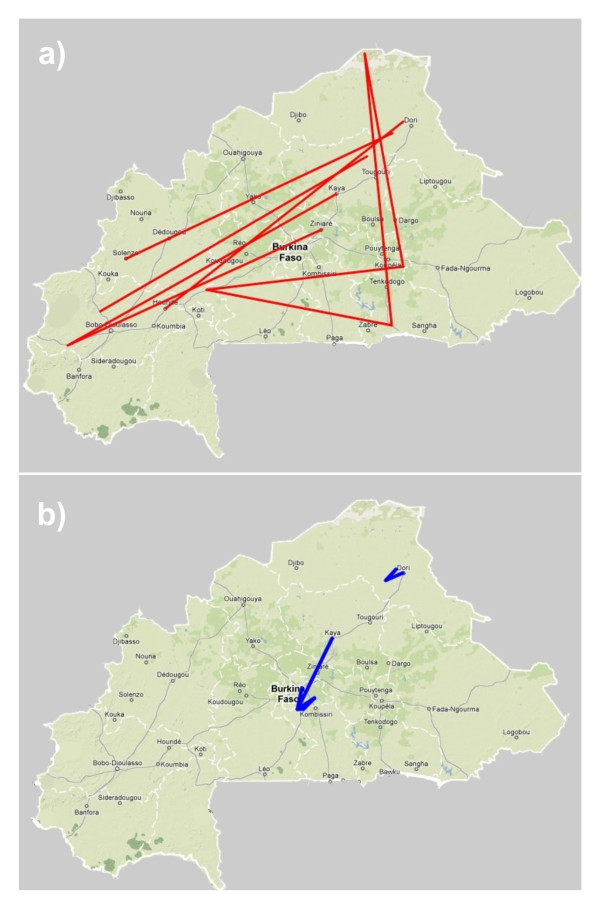
** Population Graph analysis.** Extended (Plot **a**) and compressed edges (Plot **b**) between the sampled populations of Burkina Faso goat computed using the program GENETICSTUDIO. The graph topology was exported to the freeware program GoogleEarth.

The distribution of genetic covariance among populations, as depicted in the Population Graph (Figure 4), revealed a topology consistent with increased gene flow among environmental areas. Edges connecting populations in the Population Graph are indications of significant genetic covariance between populations. The Sahel and the Sudan environmental areas of Burkina Faso were connected through a significant number of extended edges (Figure 4a), which would be consistent with the hypothesis of long-distance dispersal [26]. Conversely, compressed edges were mainly identified in the Central Sudan-Sahel environmental area (Figure 4)a. These compressed edges connected populations that were more spatially proximate than expected given the genetic covariance indicating locations of potential “genetic frontiers” [26].

The results of the partial Mantel tests carried out using the Reynolds’ genetic distance matrix and those matrices defining landscape boundaries are given in Table 2. All correlation coefficients computed were low (ranging from 0.155 to 0.233). After applying the Bonferroni correction for multiple tests, neither the Sahel (*p* > 0.05) nor the Sudan area (*p* = 0.037) limits reached the statistical significance level of 0.0125. This would suggest that: i) the goat located in the Central Sudan-Sahel area of Burkina Faso were genetically closer to the Sahelian goat than to the Dajallonké goat; and ii) the accepted limits of the environmental areas of Burkina Faso are “fuzzy” boundaries rather than ecological barriers which cannot be crossed. The more significant landscape boundary was found for the present Northern limit of trypanosome vectors (*p* = 0.008), confirming that the presence of tsetse fly influences the genetic variability assessed in Burkina Faso goat. The inclusion of the populations sampled on the road from Ouagadougou and Bobo Dioulasso within the area free of tsetse flies did not improve the results obtained (*p* = 0.046). The road may make the gene flow from North to South easier but the significance of its influence on the whole Burkina Faso goat population is questionable.

**Table 2:** Values of the statistics Z and r for partial Mantel tests assessed using the between-populations Reynolds’ genetic distance matrix and different landscape boundaries identified in the Burkina Faso territory

**Boundary**	**Z**	**r**	**p-value**
*i*) present limit of the Sahel area	3.28	0.166	0.072
*ii*) present limit of the Sudan area	2.53	0.233	0.037
*iii*) present Northern tsetse limit	3.39	0.155	0.008
*iv*) tsetse limits + human action^1^	2.97	0.167	0.046

Results were obtained after accounting for effects of a third matrix giving information on the three environmental areas of Burkina Faso (Sahel, Sudan-Sahel or Sudan) in which a given population is located. The associated probabilities were calculated by carrying out 10,000 permutations of lines or columns of one of the matrices. Note that, after applying the Bonferroni correction, each of the four partial Mantel tests carried out should be tested at a statistical significance level of 0.0125 for α = 0.05.

^1^like iii) but separating from the tsetse infested area those populations sampled on the road from Ouagadougou to Bobo Dioulasso.

## Discussion

As expected [10], the scenario analysed has a poor overall differentiation. A recent study involving 6 trypanotolerant and 3 trypanosusceptible West African goat populations sampled in 8 different countries, from Senegal to Chad, concluded that genetic differentiation in West African goat is basically due to geographic distance [27]. Morphological differences or expected different origins of the analysed goat populations did not result in high genetic differentiation at the microsatellite level [27]. This has also been shown using mitochondrial DNA markers [28]. The present study, however, captured higher genetic variability than that assessed in a previous study on Burkina Faso goat due to the higher availability of populations and samples. Traoré et al. [10], using roughly a quarter of the samples analyzed here and a consistent microsatellite set, estimated the overall differentiation of Burkina Faso goat as half that of the current study (F_ST_ = 0.035 ± 0.003 vs. F_ST_ = 0.067 ± 0.003). Therefore, although the overall genetic differentiation assessed is still moderate, the patterns of genetic variation identified in the present analysis are likely to characterise a wide genetic clinal pattern in the Burkina Faso goat stock. Partially, the moderate levels of genetic differentiation found can be explained by the characteristics of goat as livestock species. Goat is a portable food resource, particularly well adapted to harsh environmental and management conditions, that has been extensively used in human migrations and commercial trade, therefore leading to extensive genetic exchanges. In any case, the goat population of Burkina Faso is far from being homogeneous. The analysis carried out using the program Barrier showed the existence of genetic boundaries in the population that are clearly consistent with the results obtained using other methodologies (Figure 2): a) the “pure” Sahelian goat can be differentiated from the rest of the Burkina Faso goat; b) there exists genetic discontinuities in the Central and Southeast Burkina Faso; and c) the Bobo Dioulasso can have genetic differences with the other populations sampled in the Sudan area.

The assumptions on which the models implemented in the programs LEA and LEADMIX do not fit exactly with the “real world” scenario analysed here and therefore their results should be interpreted with caution. However, the results obtained in this study point in the same direction regardless the different assumptions underlying the applied methodologies. In fact, they work under different population models (i.e. LEADMIX and LEA) or they do not work under any explicit genetic model (in the case of PCA). Therefore, we can be confident in the robustness of the assessed clinal pattern.

From our results, it can be inferred that the Sahelian goat: i) is genetically different to most goat populations in the Central and Southern parts of the country (Figures 1 and 2); and ii) is in geographic expansion (see Figure 4a). The patterns of genetic variation ascertained do not follow a simple variation according to latitude. This hypothesis, which was the basis of previous studies on Burkina Faso domestic small ruminant populations [1,9,10], could only be accepted if no other factors than the progressive desertification of the country would have favoured the expansion of the Sahelian goat. The limits of the Sahel area shifted to the south from the 1970’s to present [7] but introgression of Sahelian goat genes into South Burkina Faso also varies with longitude. This fact is more evident East from the Nakambé river (Figure 3; Additional file 1).

The inheritance of goat trypanotolerance is still poorly understood [11]: trypanotolerance in small ruminants is less pronounced than in cattle and, furthermore, there exists evidence suggesting that Djallonké goat are less trypanotolerant than Djallonké sheep [see 11 for a review]. In fact, Burkina Faso sheep sampled in the Sudan tsetse infested area (Djallonké) had higher genetic differentiation with the Sahelian sheep than their goat counterparts [1,10]. Sahelian goat may be competitive with Djallonké goat in tsetse infested areas [12,13], therefore increasing the possibilities of introgression of Sahelian goat genes into Southern Burkina Faso. Although Djallonké goat should be considered as resilient rather than resistant to trypanosomosis [11], it has been suggested that Djallonké goat possess an innate ability to acquire immunity in scenarios of repetitive trypanosome infections [11,13]. This superior ability makes possible the Djallonké goat to prevail in tsetse infested areas. However, this resilience to trypanosomosis can be in risk due to a major introgression of genes of trypanosusceptible goat breeds south in West Africa [11].

Our results are consistent with previous reports made at a regional level in West Africa suggesting an increasing gene flow that goes from Sahelian goat into Djallonké goat because of progressive desertification [11]. Other local studies carried out in sheep [1] or cattle [3] support our findings as well [3].

The Sahelian goat has clearly beyond the limits of the Sahel area of Burkina Faso. The higher genetic contributions of Sahelian goat found in Eastern longitudes in Burkina Faso may be explained by the disappearance, in the absence of permanent rivers or springs, of trypanosome vectors in areas with annual rainfall beyond 800 mm [29,30]. As reviewed by Courtin et al. [14], the presence of tsetse flies east the Nakambé river constantly shifted southwards from the second half of the 20th century (see Additional file 1). Most of this area belongs to the Burkina Faso Niger basin in which no permanent rivers or springs exist. The existence of a relatively gentle flow in the southern part of the Nakambé river allow the maintenance of riverine protected forests and vegetation along its course. In this suitable habitat for tsetse flies the trypanotolerant Djallonké goat [5] may prevail. A similar scenario explains the fact that the Northeastern limit of the tsetse distribution in Burkina Faso (near the Mouhoun river loop) remains at about the same latitude since middle 20th century [14]. The Mouhoun river basin is widely recognised as an area in which riverine species of tsetse seem to be resilient to man-made changes [31-33].

However, direct human action may also underlie geographic genetic variation in Burkina Faso goat. Burkina Faso is a relatively overpopulated country with most of the population inhabiting between Ouagadougou and Bobo Dioulasso. There is a broad goat trade at these two cities. This may explain the genetic differences indentified between population 18 and the other populations sampled in the Sudan area of Burkina Faso (Figures 2 and 3). However, results from the partial Mantel tests suggest that the introgression of Sahelian goat genes into Djallonké goat using human-influenced genetic corridors has a limited influence when compared to the biological boundary defined by the northern limits of the distribution of the tsetse fly. The increase of human population is also influencing the possibilities of spreading of Sahelian goat. Courtin et al. [14] reported the limits of the expansion of the tsetse flies has been largely shifted southwards in the area surrounding the Burkina Faso capital Ouagadougou, probably due to the combined action of a decrease in rainfall and an increase in human density. Burkina Faso is experiencing considerable environmental changes as a result of an unprecedented demographic increase. At present, human population number 13,393,000 inhabitants, with a rough increase of 3 million people from the 1996 census [6]. As in other parts of Africa [30], the progressive clearing of the natural vegetation for cultivation, the introduction of domestic animals and the almost complete disappearance of large wildlife species have limited the distribution, density, dispersal and lifespan of tsetse flies significantly.

## Conclusions

As a summary, it has been shown that genetic variation in the Burkina Faso goat population follows geographic patterns. The current analysis clearly suggests that genetic variation in Burkina Faso goat: a) follows a North to South clinal; and b) is affected by the distribution of the tsetse fly that imposes a limit to the Sahelian goat expansion due to their trypanosusceptibility. Our results suggest that the most significant landscape boundary affecting genetic variation in Burkina Faso goat was the present Northern limit of trypanosome vectors, confirming that the presence of tsetse fly influences the genetic variability assessed in Burkina Faso goat. Here we showed how extensive surveys on livestock populations in developing countries can be useful to indirectly assess the major forces action on human-influenced ecosystems in a climate change framework.

## Methods

### Sampling and genotyping

Blood samples were obtained from a total of 520 reproductive individuals (220 bucks and 320 does), in 23 different villages located in the 3 environmental areas of Burkina Faso (8 belonging to the Sahel area; 10 to the Sudan-Sahel area; and 5 to the Sudan area; see Table 1 and Additional file 1). From these samples, 113 were previously available [10]. Within each village, from 3 to 10 different herds were sampled. In Burkina Faso, goat herds are usually small. Size of the sampled herds ranged between 15 and 30 individuals. Management of herds is not usually communal. Constant medium-range movement between districts in search of grazing areas leads to genetic exchanges between herds. When possible, sampling within a herd included the 2 older does and the younger buck to avoid close genetic relationships between individuals. Throughout the manuscript, the individuals sampled in a given village will be referred as populations. Eight populations were sampled in the present limits of the Sahel area ( Additional file 1; Table 1), 10 within the Sudan-Sahel area and 5 in the Sudan area. Seven sampled populations were located out of the limits of the Volta basin. Five populations were sampled between the Nakambé and Nazinon rivers and other 7 populations were under direct influence of the Mouhoun river. Nine populations were located on the main road of Burkina Faso which carries traffic from Dori to Ouagadougou and from Ougadogou to the second city of the country, Bobo Dioulasso.

Total DNA was isolated from blood samples following standard procedures [34]. A microsatellite set, including 19 markers (BM6526, BM757, BMS2626, BMS356, CSSM66, McM53, RBP3, BM8125, BMS2461, BMS975, CSRD2111, CSSM31, ILSTS005, INRA26, McM527, OarHH64, SPS115, TGLA53 and LSCV29) previously used in diversity analyses of goat [10] and sheep [1,35], was analyzed on all the individuals (see Table 1). Genotyping was performed on an Automatic Sequencer ABI 310 (Applied Biosystems, Barcelona). Data deposited in the Dryad repository: http://dx.doi.org/ 10.5061/dryad.41h46j37.

### Statistical analyses

The following parameters were computed using the program MolKin [36] (version 3.1): expected heterozygosity (H_e_), Wright’s F-statistics and raw (*A*) and ‘rarefacted’ (*A*_*(g)*_) average number of alleles per locus. Here, g was fitted to 6. Using also the program MolKin, the between-populations Reynolds’ distance (*D*_*R*_) and molecular coancestry (*f*_*ij*_). As based on a pure drift model, the *D*_*R*_ has been shown to be an appropriate measure for livestock populations with short-term divergence [37] while molecular coancestry can be interpreted as a measure of the between-populations genetic identity [21,38]. To avoid bias because of unequal sample sizes, statistical significance of the obtained values the bootstrapping method recommended by Simianer [39,40] was applied using 1000 samples with exactly 23 (the average population size) individuals per sampled population. See the User’s Guide of the program MolKin (freely available at http://www.ucm.es/info/prodanim/html/JP_Web.htm) for a detailed description of the methodologies used. In any case, statistical analyses were re-run after removing the five smallest populations (4, 5, 14, 15, and 16) from the dataset to avoid any bias due to differences in sample size (Additional file 3: Figure S1). Results were highly consistent with those obtained with the full dataset. For descriptive purposes, multidimensional scaling analysis was carried out on the genetic distance matrices using the Proc MDS of SAS/STAT^TM^ (SAS Institute Inc, Cary NC).

The program Barrier version 2.2 [23] was used to identify possible genetic discontinuities in our dataset. This program uses a Delaunay triangulation to connect the geographic locations of the sampled populations on the map and then apply a Monmonier’s Maximum-difference algorithm [22], for the identification of genetic discontinuities as follows: a) by selecting the edge of the network with the largest allocated genetic distance and using it as the starting point of the barrier perpendicular to the network boundary; and b) selecting the edge which is directly connected with the growing barrier with the largest genetic distance for the continuation of the barrier. The robustness of the computed genetic discontinuities was assessed calculating 19 between-populations Reynolds’ distance matrices using jackknifing over microsatellites.

Genetic information was also summarised via computing a Principal Component Analysis (PCA) from the correlation matrix among allelic frequencies using the Proc Factor of SAS/STAT^TM^ according to the recommendations by Cavalli-Sforza et al. [41].

The relative contributions of the Sahelian and Djallonké goat to each sampled population were assessed using the programs LEADMIX [24] and [25] LEA. Parental populations were formed pooling the individuals belonging to populations from 1 to 5 for the Sahelian population and those belonging to populations 19, 21, 22 and 23 for the Djallonké breed. The program LEADMIX [24] is a maximum likelihood method that takes into account the genetic differentiation between parental populations in the admixture calculation. LEADMIX is based on a simple model where two or more parental populations diverge from an older ancestor, and then meet during an admixture event to create a third ‘hybrid’ population. In this way, the method aims to avoid falsely assuming independent allele frequency distributions of the parental populations and any resultant bias in the admixture calculation. The program LEA [25] is based on a different demographic model where the two parental populations are assumed to be at demographic equilibrium and the allele frequencies prior to admixture are sampled from independent uninformative prior probability distributions. It also accounts for genetic drift, which is estimated through the scaled parameters t1 = T/N1, t2 = T/N2, th = T/Nh, where T is the time since the admixture event (in generations), and Ni is the effective size of population I (with I = 1, 2, h). LEA implements a full-likelihood Bayesian method, and hence provides posterior distributions for the parameters of the model, rather than point estimators. Either two or three independent runs were performed for each population, using different starting values in the parameter space, to determine whether equilibrium had been reached [42]. Each run had at least 500,000 steps together with a thinning interval of five. Also, a few longer runs (up to 1 × 10^6^ steps) were used to check for convergence.

The PCA, LEADMIX and LEA scores obtained for each population were used to construct interpolation maps drawn using the Spatial Analyst Extension of ArcView, available at: http://www.esri.com/software/arcview/. The Inverse Distance Weighted (IDW) option with a power of two was selected for the interpolation of the surface. IDW assumes that each input point has a local influence that diminishes with distance. The area of sampling of each population was used as geographic coordinates, and the six nearest neighbors were used for the calculation. Interpolation surfaces were divided into seven equal classes.

Population Graph analysis [43,44] was performed, using the program GENETICSTUDIO [45], to infer which populations may have in the past or are still experiencing gene flow. The genetic distances between populations in the network were then regressed on Euclidean distance (using a Mantel approach at the α = 0.05 significance level) to estimate isolation-by-graph-distance (IBGD) which provides an indication of two different categories of spatial genetic discontinuities. The first category consists of populations that are spatially closer than expected given their genetic covariance. In a Population Graph, the edges connecting these populations are “compressed” indicating potential locations of vicariance [26]. The second category of spatial genetic discontinuity are those populations who are spatially much further apart than expected given their genetic covariance. Here, the edges in the Population Graph are “extended” and are consistent with a scenario of long distance dispersal [26]. Data were visualised by exporting the graph topology to the freeware program GoogleEarth.

Partial Mantel test [46,47] was carried out using the program Arlequin 3.5.1.2 [48] to check for the statistical significance of the main landscape boundaries identified in Burkina Faso. In partial Mantel test, purely spatial effects on genetic differentiation are accounted for before assessing landscape effects. Here, correlation among the between-populations Reynolds’ distance matrix and different matrices defining landscape boundaries is assessed while controlling for effects of a matrix giving information on the environmental areas of Burkina Faso (Sahel, Sudan-Sahel or Sudan) in which a given population is located. Reynolds’ distance was selected due to its nice linear behaviour [37] which fits well with the Mantel test expectations [46,47] and its assumption of a pure drift model which fits well with poorly differentiated livestock breeds [37]. Following Balkenhol et al. [49], the presence of landscape boundaries was assessed via creating dummy matrices that indicated whether a population pair was separated by a boundary or not (noted in the matrix as 1 or 0, respectively; Table S2). Also following Balkenhol et al. [49], a matrix characterising environmental areas was created using a dummy variable that placed a population in a certain area not bisected by landscape boundaries (i.e. the population received a “1” in the column belonging to that area, and a “0” in all other columns; Additional file 4: Table S2). The statistical significance of the following boundaries were assessed: *i*) the present limit of the Sahel area; *ii*) the present limit of the Sudan area; *iii*) the present Northern tsetse limit in Burkina Faso [11]; and *iv*) like boundary *iii* but including in the tsetse free area those populations sampled on the road from Ouagadougou to Bobo Dioulasso (13, 17 and 18). These four analyses are expected to characterise if differentiation between the Sahelian, Mossi and Djallonké goat has genetic support (matrices *i* and *ii*), the genetic differentiation due to the presence or absence of trypanosome vectors (matrix *iii*) and the combined effect of the presence of trypanosome vectors and the human action (matrix *iv*). In all cases, p-values were obtained using 10,000 permutations. Statistical significance level of the four hypothesis tested on our dataset was assessed at α = 0.05 after Bonferroni correction.

## Authors’ contributions

This work results from the collaboration between INERA and SERIDA, starting in 2007, on the characterisation of the Burkina Faso livestock populations and the assessment of the impact of the progressive desertization of the country. AT, HHT and FG conceived the project; AT, YZ, AK, GO-S, and HHT planned and performed the sampling. AT and YZ extracted DNA; IA and AT performed most laboratory analyses; LP-P contributed to laboratory analyses; IF and FG made statistical analyses; AT, IF and FG wrote the paper. All authors read and approved the final manuscript.

## Supplementary Material

Additional file 1 **Supplementary Background.** Detailed description of the geography, environmental areas and goat populations of Burkina Faso. Click here for file

Additional file 2 Table S1. Between-populations molecular coancestry and Reynolds’ distance matrices. Click here for file

Additional file 3 Figure S1. Bidimensional scaling plots constructed using genetic distance matrices computed after removal of the five smallest populations (4, 5, 14, 15, and 16). Click here for file

Additional file 4 Table S2. Dummy matrices created to characterise the presence of landscape boundaries in the Burkina Faso territory. Click here for file
