# Supplementary material for: Ascertaining gene flow patterns in livestock populations of developing countries: a case study in Burkina Faso goat
Source: BMC Genet. 2012 May 7;13:35. doi: 10.1186/1471-2156-13-35 (PMC3413537; doi:10.1186/1471-2156-13-35)
Supplement: Additional file 1 — Supplementary Background. Detailed description of the geography, environmental areas and goat populations of Burkina Faso. [file 1471-2156-13-35-S1.pdf]

Supplementary Background for Traoré et al. (2012) BMC Genetics.

(including one figure and one table)

Figure. Map illustrating the Burkina Faso geographical scenario. The map gives the limits of the environmental areas of Burkina Faso (Barry et al. 2005; Ouadba 1997) for the period 1951-1970 (dotted black line) and for the period 1971-2000 (solid black line). The present limits of the three environmental areas (Sahel, in the North, Sudan, in the South, and central Sudan-Sahel area) are, approximately, 15° 3' N and 11° 3' N. The limits of the Volta basin (solid gree line), the three main rivers of this basin and the locations of the sampled populations (numbers are consistent with those of Table 1) are also shown. Additionally, the map illustrates the Northern tsetse limit in Burkina Faso from 1949 to 2009. Solid yellow line illustrates the northern tsetse limit in 1949, starting from the border with Mali at the 13°N of latitude to reach the border with Niger at 13° 30' N of latitude; the limits reported in 1977 (solid orange line) show a substantial shift southwards from Kaya (population 8) to the east Burkina Faso–Benin border, equivalent to 1° 30' N of latitude ; the limits in 2009 (solid red line) start at a similar latitude than those previously shown (above the Mouhoun river loop) to shift southwards South Ouagadougou (population 10) and along the Nakambé river to reach the Togo border (11° N). The dotted grey line illustrates the main road of Burkina Faso (Dori-Ouagadougou-Bobo Dioulasso; populations 4, 10 and 18).

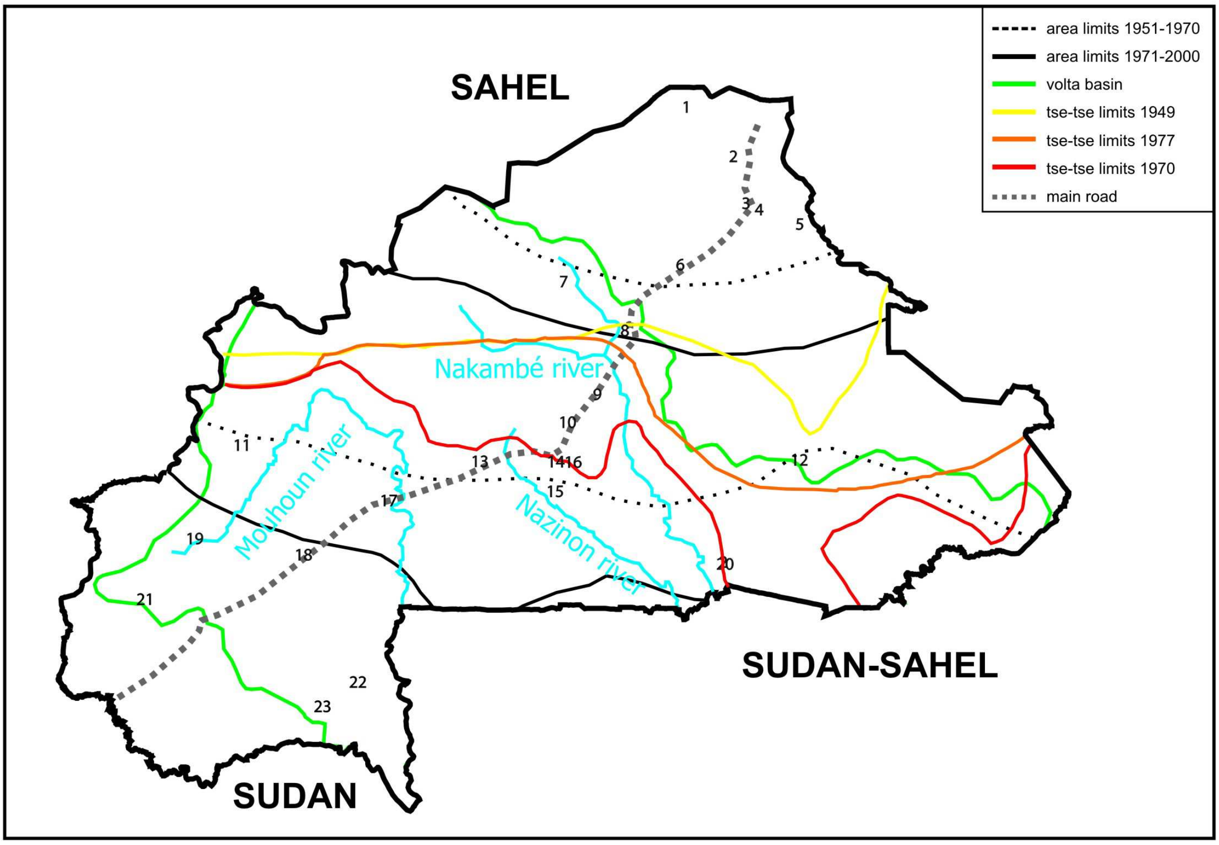

Description of the Burkina Faso Territory

Burkina Faso is a landlocked country located in the West African Sahel area. At present, human population number 13393000 inhabitants, with a rough increase of 3 million people from the 1996 census (FAO 2005). The most important cities of the country are Ouagadougou (the capital) with roughly 1.5 million inhabitants and Bobo Dioulasso, in the south of the country, with roughly 0.5 million inhabitants. A significant part of the population of the country is located in the areas surrounding and between these two cities.

Burkina Faso is a flat country with altitudes ranging from 250 to 300 m above sea level. There are no clear geographic barriers limiting the spreading of animal populations. The Volta river basin comprises the 63% of the territory of Burkina Faso (see Figure) and includes three main rivers: the Mouhoun river (formerly known as Black Volta), rising in Southwestern Burkina Faso; the Nazinon river (Red Volta), rising Northwestern Ouagadougou (the Burkinabé capital), and the Nakambé (White Volta) rising North Ouagadougou (FAO, 2005; Barry et al., 2005). The Mouhoun river is the only one of them with perennial streams. From its rise, the Mouhoun river flows northwards to Solenzo (population 11) before turning southwards (the Mouhoun loop) to flow to Boromo and the border with Ghana. Both the Nazinon and the Nakambé rivers have seasonal or intermittent streams flowing southwards into Ghana. The Northeast part of the country (30% of the territory) belongs to the Niger river basin. There are no rivers with permanent flows in this basin.

Description of the environmental areas of Burkina Faso

The climatic conditions prevailing in the country are tropical with a monomodal rainfall pattern of variable duration and increasing from north to south (from 3 to 7 months). The Burkina Faso territory can be divided in three main environmental areas regarding climate and vegetation (Ouabda, 1997; see Figure):

a) the Sahel area: this is an arid area covering the northern part of Burkina Faso (from latitude 13° 5' N to 15° 3' N, approximately) with annual rainfall < 600 mm, temperatures varying from 15 ºC to 47 ºC, and grassy, bushy, shrubby and thicket steppe vegetation, usually quite sparse, with ligneous species that may locally form penetrable bushes.

b) the Sudan-Sahel area: this is a transitional zone with regards to rainfall and temperature, covering the central part of the country (roughly from latitude 11° 3' N to 13° 5' N), with a short rainy season from June to September and very variable rainfall with average of 750 mm. per year, temperatures varying between 20 ºC and 42 ºC, and vegetation varying from North to South with better hydric conditions, from the Sahel to the Sudan savannah to tend eventually toward a clear forest in the Southwestern extreme of the domain.

c) the Sudan area: this area covers Southern Burkina Faso (latitude from 9° 3' N to 11° 3' N), shares with the Sudan-Sahel area a similar rainy season with annual rainfall > 900 mm and a predominance of woodlands and both Sudanese- and Guinean-type savannahs; temperatures are relatively low varying from 17 ºC to 35 ºC.

The droughts of the 1970s and 1980s led to a shift southwards of the limits of these environmental areas causing that most of the Volta basin in Burkina Faso is located, at present, in the Sahel and Sudan-Sahel areas (see Figure; Barry et al. 2005).

The Northern tsetse limit in Burkina Faso has shifted southwards from the middle of the 20th century to present as well (Courtin et al. 2010). In 1949, the northern tsetse limit started from the border with Mali at the 13° N of latitude, joined Kaya (population 8) at similar latitude, shifted southwards to Fada N'Gourma (population 12) and finally shifted northwards to the border with Niger at 13° 30′ N of latitude. Reports from 1977 suggested that the limits in Northwest Burkina Faso remained basically the same. However, they showed a substantial shift southwards from Kaya to the east Burkina Faso–Benin border, equivalent to 1° 30′ of latitude (approximately 145 km). The northwestern limits tsetse reported in 2009 (Courtin et al. 2010) starts at a similar latitude than those previously shown (above the Mouhoun river loop) to shift southwards following the Mouhoun river course; then it crosses South Ouagadougou (population 10) before shifting southwards again along the Nakambe river to reach the Togo border (11° N). A secondary territory with presence of tsetse flies is the South-East Burkina Faso-Togo border around the Pama protected areas (Arli).

Description of the goat population of Burkina Faso

The Burkina Faso goat population is formed by a total of 10035687 heads. This livestock represent the 69.5% of the domestic ruminants exploited in this country (ENEC II, 2004). Goat management is carried out under very harsh and traditional conditions with a significant variation in herd sizes. Goat plays a major role in maintenance of rural populations in conditions of extreme poverty in Burkina Faso and has also a major cultural importance due to its traditional use in rites and celebrations.

The rural communities of Burkina Faso recognise three main goat entities that can be considered as breeds (Traoré et al. 200, 2009):

a) the Djallonké goat breed, located in the Sudan area, is a short-eared and small-horned goat belonging to the West African Dwarf goat population, which is spread throughout the African Atlantic coastline from Guinea Bissau to Congo;

b) the Sahelian goat breed, which is the Burkina Faso representative of the long-legged goat group spread throughout the Sahel region of West Africa, from western Sudan in the east to Mauritania and Senegal in the west.

c) the Mossi breed, located in the Sudan-Sahel area of Burkina Faso, which is considered a transition breed probably nearer to the Djallonké breed (Traoré et al. 2008, 2009).

More information the goat groups to which the Burkinabé goat breeds are assigned can be found the DAGRIS database at http://dagris.ilri.cgiar.org (DAGRIS 2007).

Table. Summary of the characteristics of the three goat breeds of Burkina Faso. Body measures are described in terms of least-squared means and their standard errors while qualitative traits are described in percentage (Traoré et al. 2008).

	Sahelian goat	Djallonké goat	Mossi goat
Area of spreading	Sahel	Sudan	Sudan-Sahel
Goat group	West African Dwarf	Long-legged	Transition population linked to Djallonké goat
Trypanotolerance	Trypanosusceptible	Resilient	Moderately resilient
Ear Length[1]	16.40 ± 0.03	10.13 ± 0.06	11.51 ± 0.03
Thorax Depth[1]	25.22 ± 0.03	23.67 ± 0.05	23.07 ± 0.03
Body Length[1]	56.66 ± 0.67	51.86 ± 1.22	51.18 ± 0.67
Height at Withers[1]	61.10 ± 0.06	48.80 ± 0.11	50.36 ± 0.62
Absence of long hairs[2]	43.7	13.1	43.1
Vertical ears[2]	1.3	97.9	0.8
Ear curling[2]	73.6	0.4	26.0

[1]In cm.

[2]In percentage

References

Barry B, Obuobie E, Andreini M, Andah W, Pluquet M: Volta River Basin Synthesis. International Water Management Institute [http://www.iwmi.cgiar.org/Assessment/files_new/research]; 2005.

Courtin F, Rayaissé J-B, Tamboura I, Serdébéogo O, Koudougou Z, Solano P, Sidibé I: **Updating the Northern Limit in Burkina Faso (1949–2009): Impact of Global Change**. *J Environ Res Public Health* 2010 7**:**1708-1719.

ENEC II: *Deuxième enquète nationale sur les effectifs du cheptel*. Tome 2, 77p. Ministère de l'Agriculture et de l'Hydraulique, Ouagadougou; 2004. MED, MRA, 2004.

DAGRIS: *Domestic Animal Genetic Resources Information System (DAGRIS)*. (eds. J.E.O. Rege, O. Hanotte, Y. Mamo, B. Asrat and T. Dessie). International Livestock Research Institute, Addis Ababa, Ethiopia: 2007.

FAO: *L'irrigation en Afrique en chiffres. Burkina Faso. Enquête AQUASTAT 2005*. Food And Agriculture Organization of the United Nations, Rome; 2005.

Ouadba JM: *Elaboration d'une monographie nationale sur la diversité biologique: Collecte de données biologique, considération écologiques*. Ministère du Environment et de l'Eau, Ouagadougou; 1997.

Traoré A, Tamboura HH, Kaboré A, Royo LJ, Fernández I, Álvarez I, Sangaré M, Bouchel D, Poivey JP, Sawadogo L, Goyache F: **Multivariate analyses on morphological traits in Burkina Faso goat**. *Arch Anim Breed* 2008 **51**:588-600.

Traoré A, Álvarez I, Tambourá HH, Fernández I, Kaboré A, Royo LJ, Gutiérrez JP, Ouédraogo-Sanou G, Sawadogo L, Goyache F: **Genetic characterisation of Burkina Faso goats using microsatellite polymorphism**. *Livest Sci* 2009 **123**:322-328.
