# Supplementary material for: Ascertaining gene flow patterns in livestock populations of developing countries: a case study in Burkina Faso goat
Source: BMC Genet. 2012 May 7;13:35. doi: 10.1186/1471-2156-13-35 (PMC3413537; doi:10.1186/1471-2156-13-35)
Supplement: Additional file 2 — Table S1. Between-populations molecular coancestry and Reynolds’ distance matrices. [file 1471-2156-13-35-S2.pdf]

Table S1. Between-populations genetic identity (molecular coancestry; below diagonal) and Reynolds' distance (above diagonal) matrices. Standard deviations of the estimates are in brackets.

Population	1	2	3	4	5	6	7	8	9	10	11	12	13	14	15	16	17	18	19	20	21	22	23
1. Fadar-Fadar		0.035 (0.007)	0.039 (0.008)	0.043 (0.006)	0.037 (0.006)	0.035 (0.006)	0.030 (0.006)	0.034 (0.006)	0.038 (0.009)	0.040 (0.007)	0.041 (0.008)	0.030 (0.006)	0.036 (0.007)	0.046 (0.008)	0.049 (0.010)	0.051 (0.009)	0.041 (0.008)	0.036 (0.007)	0.053 (0.010)	0.035 (0.008)	0.050 (0.009)	0.052 (0.008)	0.058 (0.009)
2. Gorom-Gorom	0.442 (0.013)		0.030 (0.006)	0.033 (0.007)	0.038 (0.007)	0.030 (0.005)	0.024 (0.005)	0.029 (0.005)	0.039 (0.008)	0.040 (0.06)	0.037 (0.006)	0.030 (0.006)	0.033 (0.007)	0.045 (0.007)	0.049 (0.008)	0.053 (0.009)	0.036 (0.007)	0.035 (0.007)	0.039 (0.007)	0.032 (0.006)	0.044 (0.007)	0.059 (0.007)	0.056 (0.007)
3. Yakouta	0.427 (0.011)	0.389 (0.010)		0.033 (0.007)	0.035 (0.006)	0.032 (0.006)	0.025 (0.005)	0.029 (0.005)	0.034 (0.007)	0.033 (0.007)	0.036 (0.005)	0.024 (0.005)	0.030 (0.006)	0.041 (0.007)	0.046 (0.007)	0.041 (0.007)	0.031 (0.006)	0.032 (0.006)	0.048 (0.007)	0.028 (0.006)	0.047 (0.007)	0.050 (0.007)	0.052 (0.007)
4. Dori	0.424 (0.011)	0.387 (0.009)	0.375 (0.009)		0.034 (0.006)	0.029 (0.006)	0.030 (0.006)	0.036 (0.007)	0.045 (0.008)	0.041 (0.008)	0.046 (0.007)	0.031 (0.007)	0.037 (0.007)	0.048 (0.007)	0.058 (0.010)	0.057 (0.008)	0.042 (0.008)	0.041 (0.008)	0.050 (0.008)	0.038 (0.008)	0.056 (0.009)	0.061 (0.010)	0.063 (0.011)
5. Katchari	0.459 (0.010)	0.409 (0.010)	0.401 (0.009)	0.404 (0.009)		0.029 (0.006)	0.029 (0.006)	0.037 (0.007)	0.042 (0.007)	0.037 (0.007)	0.045 (0.006)	0.028 (0.005)	0.038 (0.005)	0.049 (0.006)	0.056 (0.009)	0.057 (0.007)	0.038 (0.006)	0.040 (0.006)	0.059 (0.009)	0.037 (0.008)	0.059 (0.008)	0.063 (0.007)	0.075 (0.008)
6. Tougouri	0.449 (0.012)	0.408 (0.011)	0.394 (0.009)	0.399 (0.009)	0.427 (0.009)		0.025 (0.005)	0.026 (0.005)	0.033 (0.008)	0.027 (0.006)	0.033 (0.006)	0.022 (0.005)	0.022 (0.006)	0.039 (0.006)	0.043 (0.008)	0.049 (0.009)	0.029 (0.006)	0.031 (0.006)	0.040 (0.008)	0.025 (0.007)	0.043 (0.007)	0.046 (0.007)	0.046 (0.007)
7. Yalgo	0.444 (0.012)	0.404 (0.010)	0.391 (0.010)	0.386 (0.010)	0.416 (0.010)	0.410 (0.011)		0.025 (0.005)	0.029 (0.006)	0.028 (0.005)	0.032 (0.005)	0.021 (0.004)	0.028 (0.005)	0.040 (0.006)	0.046 (0.007)	0.044 (0.007)	0.027 (0.005)	0.033 (0.006)	0.042 (0.007)	0.026 (0.006)	0.043 (0.006)	0.052 (0.007)	0.053 (0.007)
8. Kaya	0.451 (0.013)	0.408 (0.011)	0.397 (0.011)	0.391 (0.010)	0.417 (0.012)	0.419 (0.012)	0.409 (0.011)		0.031 (0.006)	0.032 (0.006)	0.03 (0.005)	0.022 (0.005)	0.027 (0.006)	0.040 (0.006)	0.043 (0.007)	0.048 (0.008)	0.028 (0.006)	0.027 (0.005)	0.042 (0.007)	0.033 (0.007)	0.042 (0.006)	0.047 (0.006)	0.051 (0.007)
9. Ziniaré	0.465 (0.012)	0.416 (0.011)	0.410 (0.010)	0.400 (0.011)	0.432 (0.010)	0.431 (0.012)	0.424 (0.011)	0.432 (0.011)		0.034 (0.007)	0.028 (0.006)	0.024 (0.006)	0.030 (0.007)	0.041 (0.007)	0.050 (0.009)	0.038 (0.007)	0.029 (0.007)	0.031 (0.007)	0.036 (0.007)	0.033 (0.007)	0.041 (0.008)	0.056 (0.009)	0.062 (0.010)
10. Ouagadougou	0.458 (0.010)	0.410 (0.009)	0.406 (0.010)	0.398 (0.009)	0.431 (0.009)	0.431 (0.011)	0.419 (0.010)	0.425 (0.011)	0.442 (0.011)		0.025 (0.006)	0.026 (0.006)	0.025 (0.005)	0.021 (0.006)	0.026 (0.006)	0.037 (0.008)	0.028 (0.007)	0.026 (0.005)	0.038 (0.008)	0.021 (0.005)	0.032 (0.008)	0.040 (0.006)	0.038 (0.007)
11. Solenzo	0.439 (0.011)	0.396 (0.010)	0.386 (0.009)	0.376 (0.008)	0.405 (0.010)	0.408 (0.011)	0.397 (0.010)	0.41 (0.012)	0.433 (0.011)	0.430 (0.011)		0.022 (0.005)	0.021 (0.004)	0.039 (0.005)	0.039 (0.007)	0.036 (0.006)	0.025 (0.005)	0.027 (0.005)	0.034 (0.006)	0.020 (0.006)	0.033 (0.006)	0.045 (0.006)	0.047 (0.007)
12. Fada N'Gourma	0.462 (0.010)	0.416 (0.010)	0.410 (0.009)	0.404 (0.009)	0.435 (0.009)	0.431 (0.010)	0.421 (0.010)	0.43 (0.011)	0.448 (0.011)	0.440 (0.010)	0.428 (0.010)		0.022 (0.005)	0.030 (0.007)	0.032 (0.006)	0.034 (0.007)	0.023 (0.005)	0.024 (0.005)	0.033 (0.007)	0.021 (0.006)	0.029 (0.007)	0.037 (0.006)	0.038 (0.006)
13. Sabou	0.463 (0.011)	0.419 (0.011)	0.410 (0.009)	0.403 (0.009)	0.431 (0.009)	0.437 (0.011)	0.420 (0.010)	0.431 (0.010)	0.447 (0.012)	0.447 (0.010)	0.435 (0.010)	0.445 (0.009)		0.026 (0.005)	0.024 (0.006)	0.035 (0.007)	0.022 (0.005)	0.022 (0.005)	0.034 (0.007)	0.019 (0.006)	0.023 (0.006)	0.034 (0.006)	0.032 (0.006)
14. Pabré	0.436 (0.010)	0.388 (0.010)	0.381 (0.009)	0.375 (0.009)	0.402 (0.008)	0.403 (0.009)	0.390 (0.010)	0.4 (0.010)	0.420 (0.010)	0.436 (0.011)	0.4 (0.010)	0.421 (0.009)	0.432 (0.009)		0.033 (0.006)	0.045 (0.008)	0.027 (0.007)	0.030 (0.005)	0.048 (0.008)	0.029 (0.006)	0.034 (0.006)	0.043 (0.006)	0.043 (0.007)
15. Saponé	0.459 (0.011)	0.409 (0.011)	0.400 (0.010)	0.389 (0.010)	0.42 (0.011)	0.423 (0.012)	0.409 (0.010)	0.423 (0.010)	0.435 (0.011)	0.455 (0.011)	0.424 (0.010)	0.443 (0.010)	0.458 (0.009)	0.432 (0.009)		0.052 (0.009)	0.03 (0.007)	0.028 (0.006)	0.051 (0.010)	0.023 (0.006)	0.034 (0.006)	0.039 (0.007)	0.036 (0.006)
16. Kamboinsé	0.416 (0.010)	0.365 (0.010)	0.367 (0.009)	0.349 (0.009)	0.379 (0.009)	0.377 (0.010)	0.372 (0.010)	0.377 (0.010)	0.409 (0.010)	0.405 (0.011)	0.389 (0.011)	0.402 (0.010)	0.408 (0.010)	0.379 (0.010)	0.397 (0.010)		0.038 (0.008)	0.039 (0.007)	0.051 (0.009)	0.037 (0.009)	0.051 (0.010)	0.056 (0.008)	0.062 (0.009)
17. Boromo	0.458 (0.012)	0.415 (0.011)	0.409 (0.011)	0.398 (0.009)	0.43 (0.010)	0.430 (0.011)	0.421 (0.011)	0.431 (0.012)	0.448 (0.011)	0.444 (0.012)	0.431 (0.011)	0.444 (0.011)	0.451 (0.011)	0.430 (0.012)	0.452 (0.012)	0.405 (0.011)		0.024 (0.006)	0.036 (0.007)	0.023 (0.006)	0.023 (0.007)	0.033 (0.007)	0.034 (0.007)
18. Bobo Dioulasso	0.453 (0.012)	0.406 (0.011)	0.398 (0.010)	0.389 (0.010)	0.418 (0.010)	0.418 (0.012)	0.405 (0.010)	0.422 (0.011)	0.437 (0.012)	0.436 (0.011)	0.419 (0.010)	0.434 (0.010)	0.441 (0.010)	0.417 (0.010)	0.444 (0.010)	0.393 (0.010)	0.44 (0.011)		0.037 (0.008)	0.022 (0.006)	0.032 (0.006)	0.036 (0.006)	0.037 (0.006)
19. Houndé	0.445 (0.009)	0.411 (0.009)	0.389 (0.008)	0.389 (0.008)	0.407 (0.008)	0.418 (0.010)	0.404 (0.009)	0.414 (0.010)	0.441 (0.010)	0.433 (0.009)	0.42 (0.009)	0.432 (0.008)	0.438 (0.009)	0.406 (0.009)	0.428 (0.008)	0.388 (0.009)	0.436 (0.010)	0.425 (0.008)		0.031 (0.008)	0.043 (0.008)	0.049 (0.009)	0.053 (0.010)
20. Bittou	0.463 (0.013)	0.418 (0.010)	0.411 (0.010)	0.402 (0.010)	0.43 (0.012)	0.433 (0.013)	0.421 (0.011)	0.424 (0.012)	0.444 (0.011)	0.450 (0.010)	0.435 (0.011)	0.445 (0.010)	0.453 (0.011)	0.427 (0.010)	0.458 (0.011)	0.404 (0.013)	0.449 (0.011)	0.440 (0.011)	0.441 (0.010)		0.023 (0.006)	0.034 (0.006)	0.030 (0.006)
21. Orodara	0.462 (0.011)	0.418 (0.011)	0.404 (0.011)	0.395 (0.011)	0.421 (0.010)	0.427 (0.012)	0.416 (0.011)	0.428 (0.012)	0.449 (0.013)	0.453 (0.012)	0.435 (0.012)	0.450 (0.011)	0.463 (0.012)	0.435 (0.010)	0.460 (0.010)	0.402 (0.011)	0.462 (0.013)	0.444 (0.012)	0.441 (0.012)	0.461 (0.011)		0.043 (0.008)	0.044 (0.007)
22. Gaoua	0.473 (0.014)	0.415 (0.012)	0.413 (0.011)	0.403 (0.012)	0.43 (0.012)	0.437 (0.013)	0.419 (0.013)	0.436 (0.012)	0.446 (0.015)	0.457 (0.012)	0.435 (0.014)	0.454 (0.012)	0.464 (0.012)	0.439 (0.012)	0.468 (0.012)	0.410 (0.013)	0.465 (0.015)	0.452 (0.013)	0.449 (0.012)	0.462 (0.013)	0.467 (0.015)		0.025 (0.005)
23. Kampti	0.456 (0.015)	0.408 (0.012)	0.400 (0.013)	0.390 (0.013)	0.406 (0.012)	0.426 (0.015)	0.407 (0.014)	0.421 (0.013)	0.429 (0.015)	0.448 (0.014)	0.422 (0.013)	0.443 (0.013)	0.455 (0.013)	0.428 (0.013)	0.460 (0.015)	0.392 (0.013)	0.454 (0.016)	0.440 (0.013)	0.433 (0.012)	0.456 (0.013)	0.456 (0.015)	0.488 (0.016)	
