# Supplementary material for: Ascertaining gene flow patterns in livestock populations of developing countries: a case study in Burkina Faso goat
Source: BMC Genet. 2012 May 7;13:35. doi: 10.1186/1471-2156-13-35 (PMC3413537; doi:10.1186/1471-2156-13-35)
Supplement: Additional file 3 — Figure S1. Bidimensional scaling plots constructed using genetic distance matrices computed after removal of the five smallest populations (4, 5, 14, 15, and 16). [file 1471-2156-13-35-S3.pdf]

Table S2. Dummy matrices created to characterise the presence of landscape boundaries in the Burkina Faso territory. Populations separated by a boundary were noted in the matrix as 1. **Matrix_A** informs on the location of a given population in a certain environmental area not bisected by landscape boundaries. **Matrix_i** characterizes the present limit of the Sahel area. **Matrix_ii** characterizes the present limit of the Sudan area. **Matrix_iii** characterizes the present Northern tsetse limit in Burkina Faso. **Matrix_iv** is like Matrix_iii but considering as tsetse free the populations 13, 17 and 18. Numbers of the populations (Pop_) are consistent with those listed in Table 1.

**Matrix_A**	Pop_1	Pop_2	Pop_3	Pop_4	Pop_5	Pop_6	Pop_7	Pop_8	Pop_9	Pop_10	Pop_11	Pop_12	Pop_13	Pop_14	Pop_15	Pop_16	Pop_17	Pop_18	Pop_19	Pop_20	Pop_21	Pop_22	Pop_23
Pop_1	1																						
Pop_2	1	1																					
Pop_3	1	1	1																				
Pop_4	1	1	1	1																			
Pop_5	1	1	1	1	1																		
Pop_6	1	1	1	1	1	1																	
Pop_7	1	1	1	1	1	1	1																
Pop_8	1	1	1	1	1	1	1	1															
Pop_9	0	0	0	0	0	0	0	0	1														
Pop_10	0	0	0	0	0	0	0	0	0	1													
Pop_11	0	0	0	0	0	0	0	0	0	1	1												
Pop_12	0	0	0	0	0	0	0	0	0	1	1	1											
Pop_13	0	0	0	0	0	0	0	0	0	1	1	1	1										

Pop_14	0	0	0	0	0	0	0	0	0	1	1	1	1	1									
Pop_15	0	0	0	0	0	0	0	0	0	1	1	1	1	1	1								
Pop_16	0	0	0	0	0	0	0	0	0	1	1	1	1	1	1	1							
Pop_17	0	0	0	0	0	0	0	0	0	1	1	1	1	1	1	1	1						
Pop_18	0	0	0	0	0	0	0	0	0	0	0	0	0	0	0	0	0	1					
Pop_19	0	0	0	0	0	0	0	0	0	0	0	0	0	0	0	0	0	1	1				
Pop_20	0	0	0	0	0	0	0	0	0	1	1	1	1	1	1	1	1	1	0	1			
Pop_21	0	0	0	0	0	0	0	0	0	0	0	0	0	0	0	0	1	0	1	1	1		
Pop_22	0	0	0	0	0	0	0	0	0	0	0	0	0	0	0	0	1	1	1	1	1	1	
Pop_23	0	0	0	0	0	0	0	0	0	0	0	0	0	0	0	0	1	1	1	1	1	1	1

**Matrix_i**	Pop_1	Pop_2	Pop_3	Pop_4	Pop_5	Pop_6	Pop_7	Pop_8	Pop_9	Pop_10	Pop_11	Pop_12	Pop_13	Pop_14	Pop_15	Pop_16	Pop_17	Pop_18	Pop_19	Pop_20	Pop_21	Pop_22	Pop_23
Pop_1	0																						
Pop_2	0	0																					
Pop_3	0	0	0																				
Pop_4	0	0	0	0																			
Pop_5	0	0	0	0	0																		
Pop_6	0	0	0	0	0	0																	
Pop_7	0	0	0	0	0	0	0																

Pop_8	0	0	0	0	0	0	0	0															
Pop_9	1	1	1	1	1	1	1	1	0														
Pop_10	1	1	1	1	1	1	1	1	0	0													
Pop_11	1	1	1	1	1	1	1	1	0	0	0												
Pop_12	1	1	1	1	1	1	1	1	0	0	0	0											
Pop_13	1	1	1	1	1	1	1	1	0	0	0	0	0										
Pop_14	1	1	1	1	1	1	1	1	0	0	0	0	0	0									
Pop_15	1	1	1	1	1	1	1	1	0	0	0	0	0	0	0								
Pop_16	1	1	1	1	1	1	1	1	0	0	0	0	0	0	0	0							
Pop_17	1	1	1	1	1	1	1	1	0	0	0	0	0	0	0	0	0						
Pop_18	1	1	1	1	1	1	1	1	0	0	0	0	0	0	0	0	0	0					
Pop_19	1	1	1	1	1	1	1	1	0	0	0	0	0	0	0	0	0	0	0				
Pop_20	1	1	1	1	1	1	1	1	0	0	0	0	0	0	0	0	0	0	0	0			
Pop_21	1	1	1	1	1	1	1	1	0	0	0	0	0	0	0	0	0	0	0	0	0		
Pop_22	1	1	1	1	1	1	1	1	0	0	0	0	0	0	0	0	0	0	0	0	0	0	
Pop_23	1	1	1	1	1	1	1	1	0	0	0	0	0	0	0	0	0	0	0	0	0	0	0

**Matrix_ii**	Pop_1	Pop_2	Pop_3	Pop_4	Pop_5	Pop_6	Pop_7	Pop_8	Pop_9	Pop_10	Pop_11	Pop_12	Pop_13	Pop_14	Pop_15	Pop_16	Pop_17	Pop_18	Pop_19	Pop_20	Pop_21	Pop_22	Pop_23
Pop_1	0																						

Pop_2	0	0																	
Pop_3	0	0	0																
Pop_4	0	0	0	0															
Pop_5	0	0	0	0	0														
Pop_6	0	0	0	0	0	0													
Pop_7	0	0	0	0	0	0	0												
Pop_8	0	0	0	0	0	0	0	0											
Pop_9	0	0	0	0	0	0	0	0	0										
Pop_10	0	0	0	0	0	0	0	0	0	0									
Pop_11	0	0	0	0	0	0	0	0	0	0	0								
Pop_12	0	0	0	0	0	0	0	0	0	0	0	0							
Pop_13	0	0	0	0	0	0	0	0	0	0	0	0	0						
Pop_14	0	0	0	0	0	0	0	0	0	0	0	0	0	0					
Pop_15	0	0	0	0	0	0	0	0	0	0	0	0	0	0	0				
Pop_16	0	0	0	0	0	0	0	0	0	0	0	0	0	0	0	0			
Pop_17	0	0	0	0	0	0	0	0	0	0	0	0	0	0	0	0	0		
Pop_18	1	1	1	1	1	1	1	1	1	1	1	1	1	1	1	1	1	0	
Pop_19	1	1	1	1	1	1	1	1	1	1	1	1	1	1	1	1	1	0	0

Pop_20	0	0	0	0	0	0	0	0	0	0	0	0	0	0	0	0	0	1	1	0			
Pop_21	1	1	1	1	1	1	1	1	1	1	1	1	1	1	1	1	1	0	0	1	0		
Pop_22	1	1	1	1	1	1	1	1	1	1	1	1	1	1	1	1	1	0	0	1	0	0	
Pop_23	1	1	1	1	1	1	1	1	1	1	1	1	1	1	1	1	1	0	0	1	0	0	0

**Matrix_iii**	Pop_1	Pop_2	Pop_3	Pop_4	Pop_5	Pop_6	Pop_7	Pop_8	Pop_9	Pop_10	Pop_11	Pop_12	Pop_13	Pop_14	Pop_15	Pop_16	Pop_17	Pop_18	Pop_19	Pop_20	Pop_21	Pop_22	Pop_23
Pop_1	0																						
Pop_2	0	0																					
Pop_3	0	0	0																				
Pop_4	0	0	0	0																			
Pop_5	0	0	0	0	0																		
Pop_6	0	0	0	0	0	0																	
Pop_7	0	0	0	0	0	0	0																
Pop_8	0	0	0	0	0	0	0	0															
Pop_9	0	0	0	0	0	0	0	0	0														
Pop_10	0	0	0	0	0	0	0	0	0	0													
Pop_11	1	1	1	1	1	1	1	1	1	1	0												
Pop_12	0	0	0	0	0	0	0	0	0	0	1	0											
Pop_13	1	1	1	1	1	1	1	1	1	1	0	1	0										

Pop_14	0	0	0	0	0	0	0	0	0	0	1	0	1	0									
Pop_15	1	1	1	1	1	1	1	1	1	1	0	1	0	1	0								
Pop_16	0	0	0	0	0	0	0	0	0	0	1	0	1	0	1	0							
Pop_17	1	1	1	1	1	1	1	1	1	1	0	1	0	1	0	1	0						
Pop_18	1	1	1	1	1	1	1	1	1	1	0	1	0	1	0	1	0	0					
Pop_19	1	1	1	1	1	1	1	1	1	1	0	1	0	1	0	1	0	0	0				
Pop_20	1	1	1	1	1	1	1	1	1	1	0	1	0	1	0	1	0	0	0	0			
Pop_21	1	1	1	1	1	1	1	1	1	1	0	1	0	1	0	1	0	0	0	0	0		
Pop_22	1	1	1	1	1	1	1	1	1	1	0	1	0	1	0	1	0	0	0	0	0	0	
Pop_23	1	1	1	1	1	1	1	1	1	1	0	1	0	1	0	1	0	0	0	0	0	0	0

**Matrix_iv**	Pop_1	Pop_2	Pop_3	Pop_4	Pop_5	Pop_6	Pop_7	Pop_8	Pop_9	Pop_10	Pop_11	Pop_12	Pop_13	Pop_14	Pop_15	Pop_16	Pop_17	Pop_18	Pop_19	Pop_20	Pop_21	Pop_22	Pop_23
Pop_1	0																						
Pop_2	0	0																					
Pop_3	0	0	0																				
Pop_4	0	0	0	0																			
Pop_5	0	0	0	0	0																		
Pop_6	0	0	0	0	0	0																	
Pop_7	0	0	0	0	0	0	0																

Pop_8	0	0	0	0	0	0	0	0															
Pop_9	0	0	0	0	0	0	0	0	0														
Pop_10	0	0	0	0	0	0	0	0	0	0													
Pop_11	1	1	1	1	1	1	1	1	1	1	0												
Pop_12	0	0	0	0	0	0	0	0	0	0	1	0											
Pop_13	0	0	0	0	0	0	0	0	0	0	1	0	0										
Pop_14	0	0	0	0	0	0	0	0	0	0	1	0	0	0									
Pop_15	1	1	1	1	1	1	1	1	1	1	0	1	1	1	0								
Pop_16	0	0	0	0	0	0	0	0	0	0	1	0	0	0	1	0							
Pop_17	0	0	0	0	0	0	0	0	0	0	1	0	1	0	1	0	0						
Pop_18	0	0	0	0	0	0	0	0	0	0	1	0	1	0	1	0	1	0					
Pop_19	1	1	1	1	1	1	1	1	1	1	0	1	1	1	0	1	1	1	0				
Pop_20	1	1	1	1	1	1	1	1	1	1	0	1	1	1	0	1	1	1	0	0			
Pop_21	1	1	1	1	1	1	1	1	1	1	0	1	1	1	0	1	1	1	0	0	0		
Pop_22	1	1	1	1	1	1	1	1	1	1	0	1	1	1	0	1	1	1	0	0	0	0	
Pop_23	1	1	1	1	1	1	1	1	1	1	0	1	1	1	0	1	1	1	0	0	0	0	0
