# Supplementary material for: Ascertaining gene flow patterns in livestock populations of developing countries: a case study in Burkina Faso goat
Source: BMC Genet. 2012 May 7;13:35. doi: 10.1186/1471-2156-13-35 (PMC3413537; doi:10.1186/1471-2156-13-35)

Figure S1 – Assessing bias due to differences in sample size.

Bidimensional scaling plots constructed using genetic distance matrices computed using a reduced dataset. The five smallest populations (4, 5, 14, 15, and 16) were removed from the dataset to avoid any bias due to differences in sample size. The complementary of the between-populations molecular coancestry matrix (1 - f_{ij}; Plot A) and the between-populations Reynolds' distance matrix (D_R; Plot A) were computed using the program MolKin (see Materials and Methods section). Results were highly consistent with those obtained with the full dataset (see Figure 1 and related pieces of text in the body of the manuscript). Populations sampled in the Sudan, Sudan-Sahel and Sahel areas are, respectively, in green squares, orange squares and blue triangles. Numbers are consistent with those listed in Table 1 for the sampled populations.

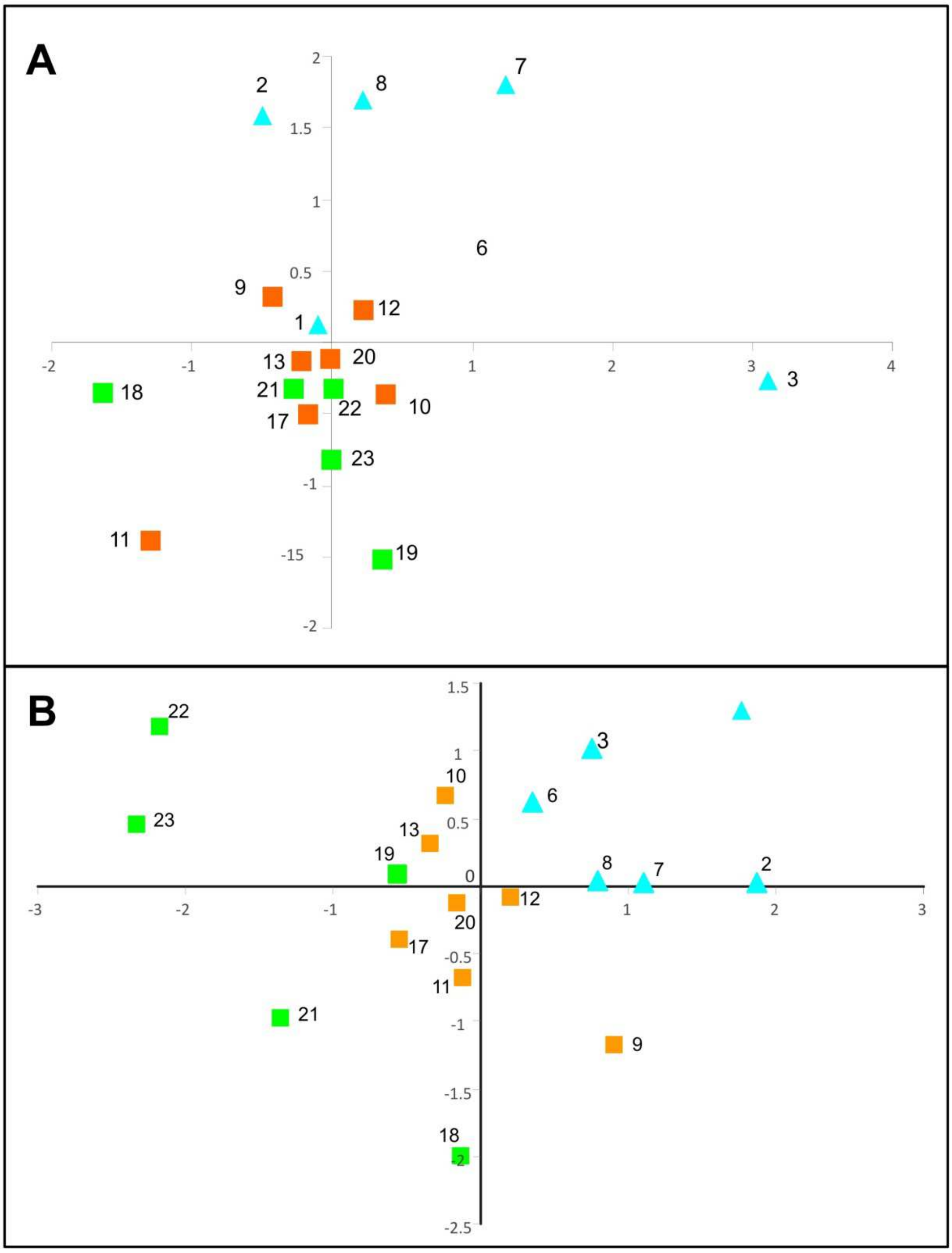

Supplement: Additional file 4 — Table S2. Dummy matrices created to characterise the presence of landscape boundaries in the Burkina Faso territory. [file 1471-2156-13-35-S4.pdf]
